# Supplementary material for: I feel like an outcast!: qualitative exploration on barriers to seek care among women with obstetric fistula in Tigray, Northern Ethiopia
Source: Reprod Health. 2025 Dec 9;22:248. doi: 10.1186/s12978-025-02186-9 (PMC12690963; doi:10.1186/s12978-025-02186-9)
Supplement: Supplementary file 2 — Supplementary Material 2. [file 12978_2025_2186_MOESM2_ESM.pdf]

I feel like an outcast! I am afraid to reach out for help: Qualitative exploration on barriers to seek care among women with obstetric fistula in Tigray, Northern Ethiopia

Liya Mamo Weldu[1,2], Haben Haileselasie[2], Znabu Hadush Kahsay[1,2], Brhane Ayele[3], Tsegay Hadgu[3], Hailay Gebretnsae[3], Hayelom Kahsay[3], Ashenafi Asmelash[6], Tesfu Alemu[7], Melaku Abrha[8], Gebrehaweria Gebrekurstose[4], Mussie Alemayehu Geberselassie[1,3,] Araya Abrha Medhanyie[1,2]

[1]School of Public Health, Mekelle University, College of Health Sciences, Mekelle, Tigray, Ethiopia.

[2]MARCH research center, College of Health Sciences, Mekelle, Tigray, Ethiopia

[3]Tigray Health Research Institute, Mekelle, Tigray Ethiopia

[4]Tigray Health Bureau, Mekelle, Tigray Ethiopia

[5]UNICEF, Tigray regional office, Ethiopia

[6]Mums for Mums, Mekelle, Tigray, Ethiopia

[7.] UNFPA, Tigray filed office, Ethiopia

[8]Mekelle Hamlin Fistula center, Mekelle, Tigray region, Ethiopia

Corresponding Author: Liya Mamo Weldu **(Email: mliya6555@gmail.com)**

Abstract

Background: Obstetric fistula, an abnormal opening between a woman's genital tract and her urinary tract or rectum. It's caused by prolonged, obstructed labor without access to timely, high-quality medical treatment. A significant number of women face barriers in accessing care, prompting this study to focus on the complicated obstacles hindering effective healthcare, and preventing timely diagnosis, treatment, and prevention.

Objectives: This study investigates the health-seeking behaviors and experiences of obstetric fistula survivors, spanning from the onset of the condition until surgical treatment is obtained.

Methodology: A qualitative study design, drawing from a phenomenological approach, was employed to explore the lived experience of a purposively selected sample of eight women with obstetric fistula and six key informant interviews which are 5 Experts and 1 Fistula ambassador/ treated. Transcription was used, entered as primary documents into Atlas. ti 9 software. Thematic categories were identified.

Results: The study identified barriers for health-care seeking and experiences of women with obstetric fistula, Women's experiences were marked by prolonged suffering, social isolation, and delayed healthcare-seeking. Many initially believed obstetric fistula was caused by spiritual punishment or witchcraft, leading them to seek help from traditional healers rather than medical facilities. Fear of shame, leaking urine, and social rejection discouraged public disclosure and prevented them from traveling to hospitals. Some women remained untreated for years due to lack of financial means or because male family members discouraged seeking help. However, survivors who accessed care typically did so with the encouragement of community health workers or NGOs, and often described the surgery as life-changing.

Conclusion: Women suffering from obstetric fistula often face complicated routes to seek treatment due to multiple factors. Primary barriers include financial difficulties, lack of awareness about the condition and its treatment, social and cultural challenges, and war. To alleviate the prolonged suffering experienced by women awaiting fistula treatment, it is crucial to increase awareness and improve access to fistula treatment center.

Key words: Obstetric Fistula, Healthcare Barriers, Tigray, Ethiopia

Introduction

World Health Organization (WHO) defined Obstetric Fistula (OF) as an abnormal opening between a woman's genital tract and her urinary tract or rectum, [1]. Beyond its clinical definition, the emotional and social toll on affected women is a critical aspect often overlooked in public health discourse. The persistent leakage, accompanied by a foul odor, significantly impacts mental well-being, causing feelings of shame, isolation, and depression. Socially, the associated stigma can strain familial relationships and lead to exclusion from community life. Studies show that women with OF often face societal discrimination, limiting access to education, employment opportunities, and overall societal participation [2, 3]. This condition is a harsh reality for three million women globally, with an additional 50,000 to 100,000 affected each year [1].

In developed nations, where obstetric care is more accessible, timely interventions and advanced healthcare infrastructure have largely eliminated OF. However, a difference arises when examining the persistent prevalence in middle and low -income countries like Asia and Africa [1, 4]. In Ghana, 1352 women per 751,205 deliveries develop OF each year with an incidence rate of 1.8 per 1000 deliveries [5]. In Ethiopia, approximately 26,000 women live with this disability with an additional 9000 new cases annually [6].

The violent conflict in Ethiopia's Tigray region has escalated the risk of OF among women [7] However, any war worsens access to healthcare and makes women particularly susceptible[7,8]. Also, conflict enhances the rates of sexual violence, there are published articles that demonstrate on the use of sexual violence as weapon and this has led to fistulas caused by direct injuries. This made worse by interferences in the provision of health services[8].

There are several global initiatives aim to eradicate OF by 2030 through prevention, treatment, and awareness. The United Nations Population Fund (UNFPA) global campaign to End Fistula, launched in 2003, has supported over 138,000 repairs across 55 countries, focusing on prevention, social reintegration, and advocacy. The WHO also targets OF eradication by 2030 within its broader maternal health agenda, promoting access to quality reproductive health services. Observed annually on May 23, the International Day to End OF raises awareness and mobilizes support for affected women. FIGO's Fistula Surgery Training Initiative addresses the lack of skilled professionals by training surgeons in fistula repair. Supporting these efforts, the UN General Assembly's 2018 resolution 73/147 advocates for accelerated action and investment

aligned with the Sustainable Development Goals. Various national strategies enhance local responses by training healthcare providers, increasing surgical access, and conducting community awareness programs, while the Global Fistula Map offers data on OF prevalence and treatment access to guide resource mobilization [9 - 12].

Early detection and intervention lead to better treatment outcomes and are often more cost-effective than delayed measures. Despite this, a significant number of women face barriers in accessing care, motivating the current study to focus on the complicated web of obstacles hindering effective healthcare, and preventing timely diagnosis, treatment, and prevention. Barriers to healthcare for obstetric fistula are multilayered and interconnected. Literature shows that socio-economic factors such as poverty, limited awareness regarding whether obstetric fistula is treatable or not lack of information on where to go for care prevent women, transportation, and geographic challenges exacerbate delays in seeking medical care [7-9, 5, 13].

In addition, cultural norms and social stigma surrounding obstetric fistula add another layer of complexity. Silence and shame prevent women from seeking prompt medical help [14], and cultural beliefs may perpetuate misconceptions about the causes and treatments of obstetric fistula, hindering effective communication between healthcare providers and affected communities [13].

In light of these multifaceted barriers, the current qualitative research seeks to study the complex challenges impeding healthcare access for obstetric fistula in Tigray, Ethiopia. By comprehending the geographical, socio-economic, and cultural dimensions of these barriers, along with other contributing factors, we aim to offer insights that guide targeted interventions and policies. This research is geared towards promoting equitable access to healthcare, thereby laying the groundwork for a comprehensive understanding to barriers for care seeking in post-conflict settings and more. Through this exploration, our goal is to unravel the complex tapestry of obstacles enveloping obstetric fistula healthcare, ultimately making a significant contribution to the global endeavor to alleviate the burden of this condition on women in vulnerable populations. Therefore, the current study aimed explore the barriers to seek care among women with obstetric fistula (OF) in Tigray, Ethiopia.

Methods and participants

Settings and Design

A descriptive phenomenological study design was used to explore the lived experiences of women with OF. The study was conducted in the three districts to obtain more representative data. Tigray is a state located in the North geopolitical region of Ethiopia.

Hamlin Fistula Ethiopia is a prominent organization dedicated to treating women suffering from obstetric fistula, Founded over 65 years ago by Dr. Catherine Hamlin and her husband, Dr. Reg Hamlin, the organization operates several facilities across Ethiopia, including the main Addis Ababa Fistula Hospital and five regional hospitals. Mekelle, Tigray being one of them and is the main setting for our research.

This study was conducted in a period of post-conflict, after ending a 3 year war in the region.

Sampling technique

A purposive sampling method was employed to facilitate the recruitment of participants who could provide rich and relevant information for the study. A total of 14 individuals participated: 8 women who were either currently living with obstetric fistula or had previously undergone surgical repair and were willing to share their experiences, and 6 key informants. The key informants included 5 experts working in maternal health and obstetric fistula care (such as healthcare providers, program officers, and policymakers) and Also, a Fistula Ambassador was also part of this participation, such people are of individuals which had relatives been treated or had went through the challenges.

Data collection tools and procedure

A semi-structured interview guide was developed by experts from multiple disciplines. The guide included main questions along with probing and follow-up questions designed to uncover new insights, clarify previously raised points, and complete the exploration of participants' opinions and experiences.

Before each interview, participants were asked to select a convenient time and private place to ensure confidentiality and comfort. Five trained investigators conducted the in-depth interviews in Tigrigna, the local language of the region. The interviews lasted between 45 and 120 minutes and were audio-recorded using recorders in a quiet setting with minimal noise.

Following data collection, the audio recordings were transcribed verbatim and translated into English. To enhance the rigor and depth of the data, investigators asked follow-up questions whenever participants' responses required further elaboration, confirmation, or clarification.

Data Quality Management

The qualitative data was collected by experienced personnel by following strict procedures, To ensure the trustworthiness of the qualitative data, experienced researchers conducted all interviews using a semi-structured guide and built rapport with participants to enhance credibility. Member checking was applied during interviews to confirm participant responses. Transferability was supported by providing detailed descriptions of participants and settings. Dependability was ensured through an audit trail documenting interview guides, transcripts, and coding processes. Confirmability was strengthened through peer debriefing and triangulation of data sources, including both fistula survivors and healthcare providers. All interviews were audio-recorded with consent, transcribed verbatim, and analyzed thematically through manual coding and regular team discussions.

Data processing and analyses

The investigators conducted in-depth interviews with 8 treated and untreated obstetric fistula survivors and 6 KII which are 5 Experts and 1 Fistula ambassador/ treated. The interviews were transcribed verbatim (in the local language) and translated into English. Each translated interview was saved as an independent file in MS Word file and imported into Atlas.ti qualitative data analysis software version 9 for coding and analysis. The investigators grouped similar codes to create categories.

Dissemination and Utilization of Results

After the data was analyzed, based on the findings obtained, conclusions and recommendations were made. Then the results of the study were submitted to the Tigray Health Bureau, Mekelle University College of Health Sciences, and Tigray Health Research Institute. In addition, the result was submitted to other stakeholders namely UNICEF, WHO, UNFPA, AMREF, Mums for Mums (a local organization proactively working on case detection and linking OF survivors for care), and Mekelle Hamlin Fistula Center. The result was presented during the dissemination of findings through the workshop. Moreover, efforts were made on the findings of the study to be published and disseminated through publications in peer-reviewed journals.

Result

Socio-demographic characteristics

Eight obstetric fistula (OF) survivors (04 treated and 04 untreated) and six key informants participated in the study. The age of the survivors ranged from 32-70 years. Nine of the ten female participants were in the childbearing age (15 to 49 years). Seven of the eight were unable to read or write. Six of the eight survivors experienced obstetric fistula at their first childbirth, (Table 1).

Table 1: Description of the participant's socio-demographic data

Participants	Age	Sex	Educational Status	Occupational status	Number of live births	Purpose for recruitment
Participant 1	34	Female	6	Milling house	3	Treated case
Participant 2	32	Female	Not educated	Farmer	3	Untreated
Participant 3	70	Female	Not educated	Farmer	7	Untreated
Participant 4	35	Female	Not educated	Housewife	2	Treated
Participant 5	44	Female	Not educated	Farmer	6	Untreated
Participant 6	35	Female	Diploma	Teacher	2	Treated
Participant 7	44	Female	Not educated	Farmer	6	Untreated
Participant 8	42	Female	Not educated	Small business owner		Treated

Table 2: Description of the participant's socio-demographic data (6KII)

Participants	Age	Sex	Educational Status	Occupational status	Number of live births	Purpose for recruitment
Participant 9	28	Male	Degree/Midwife	Midwife	-	Midwifery
Participant 10	38	Male	MSc/IESO	Employed	-	Expert
Participant 11	45	Male	Not educated	Farmer	-	Fistula ambassador/ his wife treated
Participant 12	35	Female	Degree	Employed/NGO	-	Expert
Participant 13	41	Female	Degree	Employed	-	Expert
Participant 14	36	Male	Postgraduate/Urologist	Employed		Expert

Description of Themes

Participants' ideas and reflections were organized into five major themes and their corresponding sub-themes by using an integrative deductive and inductive technique; lack of awareness, fear of disclosure, limited decision role, financial constraints and absence of care are the major themes and their sub-themes *(Table 3).*

Table 3: the themes stated in the study

Themes	Sub-theme
Theme I: Lack of awareness of cause and treatment	❖ *Not knowing the OF cause*<br>❖ *Misconception from the community on OF treatment*
Theme II: Fear of disclosure	❖ *Fear of stigma and discrimination*<br>❖ *Fear of divorce*
Theme III: Limited role on decision making	❖ *Husband's refusal*<br>❖ *No family Support*
Theme IV: Financial constricts	❖ *Lack of money for transportation*<br>❖ *Fear of the cost service*
Theme V: Impact of war on health services	❖ *Health facilities were not functional*<br>❖ *Lack of medical supplies*

Lack of awareness

Some of the participants had pointed out the lack of knowledge by most of the women in the community concerning the causes and the possibility of treating fistula noting that some believed that the only method of handling the condition is through religious means. This lack of awareness creates prejudices and stigmatization of obstetric fistula (OF). On the same note, another knowledge held within the community is that 'fistula is an incurable disease' therefore the affected mothers are either forced to stay at home or seek for other remedies such as washing with water from sacred place or consulting traditional healers which were ineffective. These perceptions even deny mothers the much-needed attention and care they would require to assist them cope with their situation.

Misconceptions about the cause of OF: Mothers expressed the cause of fistula to be related to evil spirits, indicating their poor understanding of its cause. This misperception makes them feel it is not preventable. A mother described her understanding of the condition as follows:

> *"I thought it was because of a curse or evil spirit. People said I had done something wrong or was being punished. They told me it cannot be treated in hospitals, only by traditional healers or prayer. I didn't know it was because of childbirth problems. I didn't go to the clinic because I believed nothing could be done." (34 years old, IDI participant, untreated mother)*

The misconception of treatment from the community: With the misperception that OF is caused by spirit, mother participants repeatedly reported that family members frequently discourage seeking medical treatment, explaining that it is a waste of time and money for something that is considered incurable. They strongly believe that obstetric fistula cannot be treated by healthcare providers. A mother stated as follows.

> *Family members tell you, 'Why would you go to a health facility? You will lose all your money and gold on something that's not curable.' They tell you to stay at home. If you decide to go to a health facility saying they (health providers) can do whatever with you, some people are telling you 'Why would you go there? You will not get better. They (health provider) are not God' (70 years old, IDI participant, recurred untreated woman)*

Fear of disclosure

Furthermore, participants highlighted fear of disclosure as a major barrier to seeking care. They worry that revealing their issue could result in social isolation from their community. This fear even extends to avoiding transportation due to worries about leakage, odors, and recognition. In addition, many women who have developed fistulas from sexual abuse during the aftermath of the war are reluctant to seek medical help. They fear that their spouse, family, or community might uncover the assault, leading to potential divorce and severe stigmatization, which further discourages them from pursuing necessary treatment.

Fear of stigma and discrimination*:* Participants also frequently reported that they have experienced feeling of stigmatized or outcasted because of issues such as urine and stool

incontinence, unpleasant odors, and an inability to perform daily tasks. These challenges also affected their ability to bear children heightening their fear of social isolation. They worried that these conditions would lead to their exclusion from the community. As a result, they are anxious about being recognized. Leads them to avoid using transportation to access medical care. This avoidance often causes them to withdraw from both community support and healthcare providers, A participant from mums-for-mums mentioned that women with fistula isolate themselves because of the incontinence and smell. They are afraid to use public transportation for fear of the smell. They prefer to walk on foot.

> *Due to the bad odor from the urine and stool, they don't want to socialize. Most of them prefer to walk on foot. Because of the smell and consistency, they don't want to get in a car and go to the hospital. The leakage is day and night. Even if they want to socialize, the bad smell doesn't let them (female health provider, mums for mums)*

Another participant *mother also explained the challenges of socializing with others as;*

> *Urine and stool incontinence; it is the worst illness! You can't socialize* [with others]*. You smell bad. It is the worst! It makes you disabled! It makes you below everyone! You are left alone! It makes me feel like an outcast. I'm afraid to reach out for help! (70-years-old untreated-mother*)

Fear of divorce: Another barrier mentioned by participants was when the fistula results from sexual violence women find it hard to seek care about the fear of their spouse, family, or community finding out about the violence. Furthermore, it prevents them from seeking the necessary medical attention. The consequences of divorce, stigma, and discrimination appear large, forcing these women to conceal their pain and suffering. Their condition is discovered when they visit a health facility for unrelated illnesses. One of the health care providers stated the challenges very well as follows.

> *They are troubled to speak about it because they are scared, they might get divorced. When they are asked the cause, they know it but they don't tell you. They can't resist the pressure from the community. They visit health facilities for other illnesses but not for fistula or POP. They are being identified coincidentally. (Male Health care provider)*

Another participant also stated it as;

> *It is difficult for them to tell that they were raped, because of the fear of divorce. They take time and then it gets complicated. You find them after searching and educating them. Because of our culture, they don't tell you that they are raped. They may even get divorced. They faced a lot of problems. They get discriminated against. It makes it difficult. They develop infections and other related things. Most of them don't eat to control the urine and stool. As a result, they become malnourished. It took time. You can think of the time taken until the war stopped (Female health providers, mums for mums)*

Limited decision-making power: The participants frequently explained that the limited support from their spouse in taking care of their children makes it harder to leave and seek treatment for OF leaving their responsibility behind. Further, the mother's limited role in decision-making also makes it difficult for them to seek care. When it decides against going to treatment, the mother has less say in it and will be hugely influenced by the husband's decisions,

Husband's refusal: Even when mothers know that treatment is free, they still face difficulties in accessing care as the decision to seek treatment often hinges on the husband's approval. Participants uncovered that the husband tends to refuse to allow the mother to seek care about the gap in domestic work if she is going to seek care. A participant who had a fistula for 6 years and is now waiting to get treated in the Hamlin hospital stated one of the barriers to seeking care was her husband's refusal as follows.

> *"I told him I wanted to go for treatment. He said, 'Who will take care of the children and the animals?' He told me to wait until he decides. I waited for months. It was never the right time for him." (44 years old, untreated woman)*

No Family support: The mothers who participated in the study survivors mentioned that their responsibility, including looking after the children and handling numerous household tasks, both inside and outside the home, often with little to no assistance from other family members. This extensive set of duties created significant barriers to seeking treatment, as the mother may need to defer her health needs to maintain her other responsibilities, resulting in delays or not getting necessary care. As one woman explained the challenges as follows

> *There is a woman with a fistula in our 'tabia' (Village), I asked her to come with me. She told me she couldn't leave the house unaccompanied. She told me she would go when it was spring. She even scolded me for going this time of the year (44 years old, untreated participant)*

Financial constricts: The cost of care itself was another significant factor preventing these women from seeking the necessary medical attention. Most women perceived the treatment as expensive and it can't afford, additionally, transportation costs were cited as a major barrier to accessing healthcare services. Many mothers explained that they simply could not afford the cost of public transportation to and from the health facility, as most of the health facility that gives the service is distance from their home, Without the financial means to cover these expenses, mothers found themselves caught in a cycle of limited access to healthcare, further exacerbating their already precarious situations.

Lack of money for transportation. Participants expressed difficulty in affording the bus fare required to reach a health facility. Mothers described a significant challenge in seeking treatment due to the considerable distance between their homes and the nearest healthcare facility. They noted that reaching the facility requires transportation, which is often expensive. This financial burden makes it difficult for them to afford the necessary travel. One participant's mother stated as follows.

> *There is a problem in transportation. Even though there are many cars, the cost is high. If I have no money for transportation, how can I go? Family don't give you money. They don't support you (70 years old, recurred untreated woman)*

Fear of cost service: Even though some participants were aware that they could be cured with the right treatment, they did not seek care because they believed it would be too expensive. Most mothers are not informed about the actual cost of treatment and avoid going to healthcare facilities due to fear that the service will be costly, leaving them concerned about not having enough money to cover the expenses. A participant who lived with fistula for 6 years did not seek care because she didn't know the treatment was free.

> *I know a woman who had fistula and got treated. She was rich, and her husband supported her. She told me I should go too, but I had no money and no one to help me. I didn't know the treatment was free. That's why I didn't try. It was only after talking to health workers that I learned even the transport is covered. Now I'm ready to go to Mekelle (44 years old, untreated, IDI participant)*

A mother who lost her source of income due to war stated she couldn't seek care because of a lack of money. It is also difficult to ask your family to pay for your treatment.

> *It will be difficult if I get an operation. I am going to bother her (sister). I am convincing myself it is enough if she provides food and clothes for me and my son. I know I can get better if I get treated. But I don't have the money. If I had it, I wouldn't have stayed this long (32 years old, untreated, woman)*

Impact of war on health services

The war has caused widespread destruction and dysfunction across health facilities in the region. Many of these facilities were repurposed as battlegrounds, leading to extensive damage. The remaining operational health facilities face severe shortages of medical supplies and healthcare professionals. As a result, these facilities are unable to adequately serve mothers seeking treatment, forcing them to cope with untreated fistulas and other health issues. This situation has left many mothers without the necessary medical care and support they need.

***Health facilities were not functional**:* During the war, most health facilities were closed and unable to provide services. Additionally, mothers avoided seeking treatment at these facilities due to fears of being caught by soldiers. KII participant revealed during the war, health facilities were closed, mothers feared to go to health facilities because of active war, and mothers gave birth in caves. This led to an up surging in fistula.

> *During the war health facilities were closed. This was due to fear to visit health facility. Mothers may also fear to go to health facility. Most of health facilities were non-functional. Even this facility was shut down. Mothers, who delivered during*

that time, didn't get medical service. Health providers were displaced. Most mothers gave birth in a cave. I think this all led to an up surging in fistula (28 years old, Health care professional, Female)

Lack of medical supplies: The participants specified that severe shortages of medical supplies at health facilities they have at their nearest challenge OF survivors to seek care. Despite health facilities being open and staffed with healthcare providers, the lack of essential supplies prevented them from delivering the necessary care to mothers seeking treatment by the ability of healthcare providers to offer effective treatment. A mother who developed a fistula after getting raped by soldiers and sought care during the war remembers the incident like this

There was one health provider and she was called from her home. There was no examination. That was because they (soldiers) were still there. The health provider's name was Z She was the one who assisted my delivery. She knows my condition. My brother called her from her home. He told her that I had worsened. The facility was full of broken glasses. She told us to be careful so we don't hurt ourselves. There was no medicine. She would have referred me. She would have sent me to a hospital or give me medicine or pads. My brother asked her what to do and she told us maybe we should try a private clinic (32 years old, untreated woman)

Discussion

This study reveals that both obstetric fistula survivors and service providers perceive multiple intertwined barriers affecting health-seeking behaviors. Survivors experienced prolonged suffering characterized by stigma, fear, and misconceptions about the causes and treatability of the condition, which delayed their access to care. Service providers highlighted systemic challenges such as inadequate health infrastructure, lack of trained personnel, and limited community awareness that exacerbate these delays. Both groups emphasized the critical role of community health workers and NGOs in bridging gaps by encouraging care-seeking and providing support. Together, these perspectives underscore the need for integrated interventions addressing cultural, social, and structural factors to improve timely access to obstetric fistula treatment.

Our findings have shown that both the causes and treatability of obstetric fistula (OF) are unknown to the majority of affected women and hold culturally engrained beliefs that OF is caused by

witchcraft, evil spirits, sin, punishment by God, or retribution. These views show a lack of understanding of medical practice, and contribute to the belief that OF is incurable, and therefore women with the disease should not seek treatment. There is more extensive literature about similar misunderstandings regarding OF from Uganda, Ethiopia, and India, the women described OF as divine retribution, witchcraft, or misfortune and, thus, preferred native remedies to medical ones [15–18]. Such deep-rooted misconceptions play the role of a strong barrier for women to consider a disease originating from a spiritual realm to be beyond the reach of conventional medicine and thus opt for traditional remedies only. It is for this reason that cultural beliefs and misconceptions are amongst the internal factors that hinder early access to healthcare or corporate business operations, hence affecting recovery and quality of life. Unlike the present study's findings, there are community-based awareness-raising procedures such as the Ethiopian health extension package. Thus, there is the need to step up awareness creation counter strengths the mentioned misconceptions and categorically and more effectively state that OF is a treatable condition. Culturally targeted health promotion efforts, informational sessions in communities, and engaging local decision-makers are culturally safe approaches that, if designed to counter false beliefs about these illnesses, could therefore be very powerful in changing the client's handling behaviors. Mainly, such interventions as community awareness campaigns, and enhancing access to care could play an important role in improving the awareness of OF's causes and ensure early access to medical care, fixing one of the major barriers toward adequate healthcare access in such environments [19,20].

Research from Uganda, Ethiopia, and India reveals that many women attribute the cause of obstetric fistula (OF) to supernatural forces, divine punishment, or moral failings. For instance, a study in Uganda found that women often believed their condition was caused by supernatural forces or moral lapses, which led them to pursue traditional healing methods rather than medical care [15]. Similarly, in Ethiopia, women commonly associated OF with spiritual causes or divine retribution, opting for traditional healers over clinical treatment [16]. Additionally, studies in Ethiopia and India reported that limited awareness of the medical causes of OF led women to attribute their condition to bad luck or spiritual factors, further driving them toward cultural remedies instead of evidence-based interventions [17,18]. Such misconceptions delay timely access to healthcare, exacerbating the women's suffering and complicating recovery. These findings

underscore the critical need to address cultural beliefs and improve health literacy to encourage effective healthcare-seeking behavior for OF [19, 21].

Perceived public and healthcare givers' understanding is related to stigma acts as one of the biggest hurdles among women with obstetric fistula (OF) from accessing appropriate healthcare. This work also emphasized that women with OF often apprehend social exclusion and being social outcasts for paring more disclosure of OF, believing that disclosure of this condition will only exacerbate prejudice and lead them to exclusion from society by their communities. This fear is made worse by concerns of stains, soaking and smell which makes many women avoid buses and any other social activities. Individuals with OF particularly women bear high levels of stigmatization and fear of being judged based on such symptoms, which dissuades them from seeking appropriate medical attention [22-25]. Incorporating results from preceding Ethiopian, Malawian, Tanzanian, and Nigerian researchers with OF exposed that women were deceived by stigmatization and worries of being judged due to these signs and avoided seeking medical help [22-26].

Our study aligns with findings from Ethiopia's Gonder, Tanzania, and Malawi, which consistently demonstrate that women with obstetric fistula (OF) face significant barriers to accessing care due to limited decision-making power within their households [27, 28, 29]. Studies show that healthcare decisions are often influenced by husbands or other family members, who may prioritize household responsibilities or financial constraints over the woman's health needs. This limitation in autonomy forces women to delay or forgo essential treatment, further exacerbating their condition. The impact of low decision-making power is substantial, as it restricts women from independently allocating resources, like time and money, for necessary medical care, leading to significant delays or avoidance of treatment.

When compounded with a lack of family support, this issue becomes an even stronger barrier. Women's decisions to seek care often require not only their agency but also approval and encouragement from family members, who may otherwise discourage such actions due to misconceptions about OF or resource concerns.

These dynamic underscores the need for policies and interventions that empower women's health-related decision-making. Economic interventions, such as incentive-based programs or conditional cash transfers, could increase women's financial independence, enabling them to seek care without

needing family approval. Additionally, programs that educate families on the importance of timely treatment for OF could foster supportive home environments, which are crucial for women's access to healthcare. By strengthening both women's agency and family involvement, these measures could significantly improve healthcare access for women with OF [30,31].

Lack of finances was regarded as one of the critical challenges affecting access to OF care because participants in this study acknowledged the high cost of accessing transportation and health care as some of the challenges. This is in concordance with other studies conducted across Africa, where high costs linked with travel and health services deter women from seeking OF treatment despite being offered free health care services regarding direct medical treatments [16, 29]. A lot of the additional expenditures, including transportation and lost wages, deter women from seeking care and also exacerbate the existing disadvantage for the economically vulnerable. Another study conducted in Nigeria also supports this problem, where women with fistula report up to 50% increase in economic adversity due to job loss which indicates the cost of OF to affected women and families [32]. It is vital to eradicate these financial difficulties, which implies calling for inexpensive means of transport, neighborhood healthcare services, or financial aid programs that may help the women with OF to afford both, direct and indirect treatment expenses [31].

In our study we have found that absence of functional health facilities, exacerbated by conflict and infrastructure damage, has significantly impacted the availability of care for obstetric fistula. The destruction and dysfunction of health facilities, coupled with shortages of medical supplies, have left many women without the necessary medical attention.

These in lines with a study In the Afganistan, reported that the war had led to the closure and destruction of many health facilities, creating significant barriers to accessing care for obstetric fistula. The study emphasized that the lack of functional facilities and medical supplies prevented effective treatment and exacerbated the suffering of affected women [33].

Similarly, a study in Ethiopia's Amhara region highlighted that ongoing conflict and infrastructure damage had led to severe shortages of medical supplies and non-functional health facilities[34]. This situation significantly impacted the ability of women to access timely and effective treatment for obstetric fistula.

Conclusions:

Many barriers influence the ability of women in Tigray to seek treatment for obstetric fistula, including knowledge, social, economic, decision-making, and physical barriers. Due to cultural perceptions of what is considered a curable or treatable condition, most women give preference to home remedies as opposed to seeking much-needed medical attention. In addition, there is a persistence of the barriers since war and conflicts affects access to and availability of healthcare and results in additional costs to the already vulnerable women who often have limited family and societal support. Policymakers should start culturally sensitive awareness programmes aimed at correcting the misconceptions that would jeopardize women's chances of seeking treatment for this condition, policy interventions as well as modifications should be envisaged. These measures can include increasing the confidentiality education for Health Care Workers (HCWs), increasing architectural features for more discreet patient maneuvering, and making direct patient privacy guidelines for women with OF. Similar measures, within the guideline of other successful models and avoiding lessons from other contexts, would help minimize less, decreasing barriers rooted in stigma which would in turn make squeaky for women affected passionate for timely, hospitable care without threat of identification. Henceforth, Technical quality shall also be enhanced especially on the health sectors with an emphasis on facilities that are affected by conflicts with a view of providing staff and equipment for emergency obstetric care services [35]. There is need to remove the economic barriers and means that can help include: provision of subsidies to cater for transport and treatment costs. More so, the policies which support women, to be more independent in the decisions regarding their health care would allow many women to get treatment without restrictions from the society or their families. Obstetric fistula survivors, the community, NGOs and the healthcare providers should ensure that doctors and nurses conducting counseling and treatment for obstetric fistula are culturally appropriate without stigma. Making more mobile clinics and community health centers would help to reach people who cannot get to hospitals often. That would also educate community heads to honor all pregnancies and facilitate early reporting of obstetric complications and availabilities of treatments.

Declarations

Acknowledgments

Our appreciation goes to the participants for their participation, as well as the data collectors and supervisors for their commitment and integrity to generate the data. We also thank Mums for Mums (MfM) and Hamlin fistula center Mekelle branch for their financial management and technical support the data collection respectively.

Funding

UNICEF Ethiopia WHO, AMREF Health Africa and UNFPA contributed additional fund for the study

Availability of the data and materials

The tools used for the study and the data that support the findings in the study are fully available through the authors.

Authors' contribution

MAG, LMW, ZHK, BA, HG, TH, HK, AAM, GG, HH, MA, AA, and TA conceived the study and designed the study design. All authors led the data collection process and ensure data quality. LMW and ZHK coded and analyzed the data. LMW drafted the first draft, and ZHK and AAM reviewed the first draft. All authors reviewed the second and third versions of the manuscript and approved the final draft.

Ethics approval and consent to participate

Ethical approval for the study was obtained from the Institutional Review Board (IRB) of the Tigray Health Research Institute (THRI) with a reference number of THRI/4031/1099/15. A letter of support was received from Tigray Health Bureau (TRHB). Study participants were informed about objectives of the study and verbal informed consent was sought from each participant.

Consent for publication

Not applicable

Competing interest

The authors declare that they have no competing interests.

Reference

1. *Asiedua E, Maya E, Ganle JK, Eliason S, Ansah-Ofei AM, Senkyire EK, et al. Health-seeking experiences of women with obstetric fistula: a qualitative study at two fistula centres in Ghana. BMJ Open. 2023 Aug 17;13(8).*
2. *Baker Z, Bellows B, Bach R, Warren C. Barriers to obstetric fistula treatment in low-income countries: a systematic review. Vol. 22, Tropical Medicine and International Health. Blackwell Publishing Ltd; 2017. p. 938–59.*
3. *Daniyan AB, Uro-Chukwu H, Obuna J, Mighty-Chukwu I, Yakubu E, Daniyan O. Reasons for delay in accessing free treatment of obstetric fistula in South-East Nigeria - A qualitative study. Afr J Reprod Health. 2022 Dec 1;26(12):23–31.*
4. *Chimamise C, Munjanja SP, Machinga M, Shiripinda I. Health seeking behaviors of women living with obstetric fistula in Zimbabwe: A qualitative cross-sectional study. Soc Work Public Health. 2021;36(5):548–57.*
5. *Lyimo MA, Mosha IH. Reasons for delay in seeking treatment among women with obstetric fistula in Tanzania: A qualitative study. BMC Womens Health. 2019 Jul 10;19(1).*
6. *Nambala Tembo N, Maluwa A, Odland Professor J, Katowa Mukwato P, Namukolo Kwaleyela C, Mumba Zulu J, et al. Barriers in seeking fistula repair services among women with obstetric fistula in Zambia: The case of Muchinga, Luapula, Eastern and Southern Provinces. ~ 13 ~ International Journal of Midwifery and Nursing Practice [Internet]. 2020;3(1):13–20. Available from: http://www.nursingpractice.net*
7. *Gesesew HA, Berhe KT, Gebretsadik S, Abreha M, Haftu M. Fistula in war-torn Tigray: a call to action. International journal of environmental research and public health. 2022 Nov 30;19(23):15954.*
8. *Gesesew H, Berhane K, Siraj ES, Siraj D, Gebregziabher M, Gebre YG, et al. The impact of war on the health system of the Tigray region in Ethiopia: An assessment. BMJ Glob Health. 2021 Nov 23;6(11).*
9. *Obstetric Fistula. Guiding principles for clinical management and programme development. https://www.afro.who.int/sites/default/files/2017-06/mps%20Fistula2.pdf*
10. *International Day to End Obstetric Fistula 3 May 2024. https://www.unfpa.org/events/international-day-end-obstetric-fistula*
11. *Slinger G, Trautvetter L. Addressing the fistula treatment gap and rising to the 2030 challenge. International Journal of Gynecology & Obstetrics. 2020 Jan;148:9-15.*
12. *International Day to End Obstetric Fistula. 23 May. https://www.un.org/en/observances/end-fistula-day*

13. *Varia N, Human Rights Watch (Organization). "I always remember that day" : access to services for survivors of gender-based violence in Ethiopia's Tigray region. 94 p.*

14. *Cowgill KD, Bishop J, Norgaard AK, et al. Obstetric fistula in low-resource countries: an under-valued and under-studied problem–a systematic review of its incidence, prevalence, and association with stillbirth. BMC Pregnancy Childbirth. 2015;15(1):193. doi:10.1186/s12884-015-0592-2*
15. *Nalubwama, H., El Ayadi, A. M., Barageine, J. K., Byamugisha, J., Kakaire, O., Obore, S., ... & Miller, S. (2020). Perceived causes of obstetric fistula and predictors of treatment seeking*

among Ugandan women: insights from qualitative research. African journal of reproductive health, 24(2), 129-140.)

16. *Bashah DT, Worku AG, Mengistu MY. Do community members are aware of obstetric fistula? A community based cross sectional study, Dabat district, Northwest Ethiopia. Res. J. Soc. Sci. Manag. 2018 Dec:37-50.*
17. *Feyisa W. Factors associated with knowledge and the misconception of obstetric fistula in Northwest Ethiopia. J. Women's Health Care. 2021;10:556.*
18. *Singh S, Thakur T, Chandhiok N, Singh MK, Dhillon BS. Perceptions and experiences of women seeking treatment for obstetric fistula. Birth. 2017 Sep;44(3):238-45.*
19. *Nwala, E.K., Nwaigwe, C., Sripad, P. et al. Exploring awareness of obstetric fistula in Eastern and Northern Nigeria: perceived causes, symptoms, and availability of treatment services. glob health res policy **7**, 29 (2022). https://doi.org/10.1186/s41256-022-00264-0*
20. *Asiedua E, Maya E, Ganle JK, Eliason S, Ansah-Ofei AM, Senkyire EK, Adanu R. Health-seeking experiences of women with obstetric fistula: a qualitative study at two fistula centres in Ghana. BMJ open. 2023 Aug 1;13(8):e064830.*
21. *Holme A, Breen M, MacArthur C: Obstetric fistulae: a study of women managed at the Monze Mission Hospital, Zambia. BJOG. 2007, 114 (8): 1010-1017. 10.1111/j.1471-0528.2007.01353.x.*
22. *Changole J, Thorsen VC, Kafulafula U. "I am a person but I am not a person": experiences of women living with obstetric fistula in the central region of Malawi. BMC Pregnancy and Childbirth. 2017 Dec;17:1-3.*
23. *Swain D, Parida SP, Jena SK, Das M, Das H. Obstetric fistula: a challenge to public health. Indian Journal of Public Health. 2019 Jan 1;63(1):73-8.*
24. *Abubakar FU, Nasiru MA. The Perceived Impact of Low-Stigma on Health-Seeking Behavior among Women with Obstetric Fistula in North-West Nigeria.)*
25. *Adefris M, Abebe SM, Terefe K, Gelagay AA, Adigo A, Amare S, Lazaro D, Berhe A, Baye C. Reasons for delay in decision making and reaching health facility among obstetric fistula and pelvic organ prolapse patients in Gondar University hospital, Northwest Ethiopia. BMC women's health. 2017 Dec;17:1-7.*
26. *Derso EA, Ayalew S, Eshete A, Wale M. Determinants of time to recovery from obstetric fistula by using the data of university of Gondar teaching hospital fistula center, Gondar–Ethiopia: A parametric survival regression analysis. Cogent Medicine. 2020 Jan 1;7(1):1816259*
27. *den Hollander GC, Janszen EW. Obstetric fistulas in Uganda: scoping review using a determinant of health approach to provide a framework for health policy improvement. BMC pregnancy and childbirth. 2020 Dec;20:1-8.*
28. *Mselle LT, Kohi TW. Healthcare access and quality of birth care: narratives of women living with obstetric fistula in rural Tanzania. Reproductive health. 2016 Dec;13:1-9.*
29. *Dennis AC, Wilson SM, Mosha MV, Masenga GG, Sikkema KJ, Terroso KE, Watt MH. Experiences of social support among women presenting for obstetric fistula repair surgery in Tanzania. International journal of women's health. 2016 Sep 6:429-39.*
30. *International Day to End Obstetric Fistula. 23 May. https://www.un.org/en/observances/end-fistula-day*
31. *Scott A, Liu M, Yong J. Financial incentives to encourage value-based health care. Medical Care Research and Review. 2018 Feb;75(1):3-2.*

32. *Umoiyoho A J, Inyang-Etoh E C, Abah G M, Abasiattaim A M, Akaiso O E. 2011. "Quality of Life Following Successful Repair of Vesicovaginal Fistula in Nigeria." Rural Remote Health 11 (3): 1734.*
33. *Yusufi MO, Fanning E, Bhatta MP. Determinants of obstetric fistula in Afghanistan: An analysis of the Demographic and Health Survey 2015. International Journal of Gynecology & Obstetrics. 2022 Oct;159(1):213-22.*
34. *Kodo TK, Kidie AA, Merecho TH, Tiruneh MG, Yayeh BM, Getaneh BA, Demesie AM, Wendimagegn ZS. The Impact of Armed Conflict on Services and Outcomes Related to Maternal and Reproductive Health in North Wollo, Amhara, Ethiopia: A Qualitative Study. International Journal of Women's Health. 2024 Dec 31:1055-66.*
35. *UNFPA E. Obstetric fistula needs assessment report: Findings from nine African countries. UNFPA, EngenderHealth. Retrieved January. 2003;9:2019.*
